# Three Ventricular Assist Devices (True TriVAD) as Bridge to Durable Biventricular Support (BiVAD) in LVAD Patient With Severe Aortic Regurgitation

**DOI:** 10.1111/aor.70072

**Published:** 2025-12-01

**Authors:** Nikolaos Cholevas, Felix Schoenrath, Nicolas Merke, Denis Fries, Marcus Mueller, Johanna Mulzer, Evgenij Potapov

**Affiliations:** ^1^ Department of Cardiothoracic and Vascular Surgery German Heart Center of the Charité Berlin Germany

**Keywords:** aortic regurgitation, LVAD, RVAD

## Abstract

A Left Ventricular Assist Device (LVAD) patient developed severe aortic regurgitation (AR), leading to right heart failure and multi‐organ dysfunction (MOD). Temporary Right Ventricular Assist Device (tRVAD) support caused left ventricle (LV) overload and pulmonary edema. Implantation of an Impella 5.5 successfully unloaded the LV and stabilized the patient, enabling durable RVAD implantation with surgical aortic valve replacement (SAVR). The case highlights the importance of early AR recognition and tailored mechanical circulatory support.

## Graphical abstract

An LVAD patient with severe aortic regurgitation (AR) developed right heart failure and multi‐organ dysfunction (MOD). tRVAD support caused LV overload and pulmonary edema. Impella 5.5 implantation successfully unloaded the LV and stabilized the patient, enabling durable RVAD implantation with surgical aortic valve replacement.

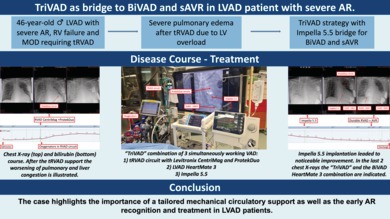

**Abbreviations**
AF: atrial fibrillation
ARDS: acute respiratory distress syndrome
DCM: dilated cardiomyopathy
ECG: electrocardiogram
LCOS: low cardiac output syndrome
LVEDD: left ventricular end‐diastolic diameter
MCS: mechanical circulatory support
MOD: multi‐organ dysfunction
TAVI: transcatheter aortic valve implantation
VC: vena contracta

## 1 Case Report

A 46‐year‐old male, 5.5 years on HeartMate 3 (HM3, Abbott, USA) support for dilated cardiomyopathy (DCM), presented with cardiac decompensation. Labs revealed worsening renal function (urea: 53.8 mg/dL, creatinine: 1.5 mg/dL), liver congestion (bilirubin: 2.2 mg/dL), and elevated NT‐proBNP (5912 pg/mL). The electrocardiogram (ECG) demonstrated new‐onset atrial fibrillation (AF) with a rapid ventricular response. From the past history, 1.5 years ago an outflow graft (OG) obstruction was successfully treated with two Bentley BeGraft stents (14 × 59 mm). At the time of the LVAD implantation, no structural or functional aortic valve (AV) dysfunction was detected. Five months ago, the echocardiography showed moderate AR and preserved right ventricular (RV) function.

**FIGURE 1:**
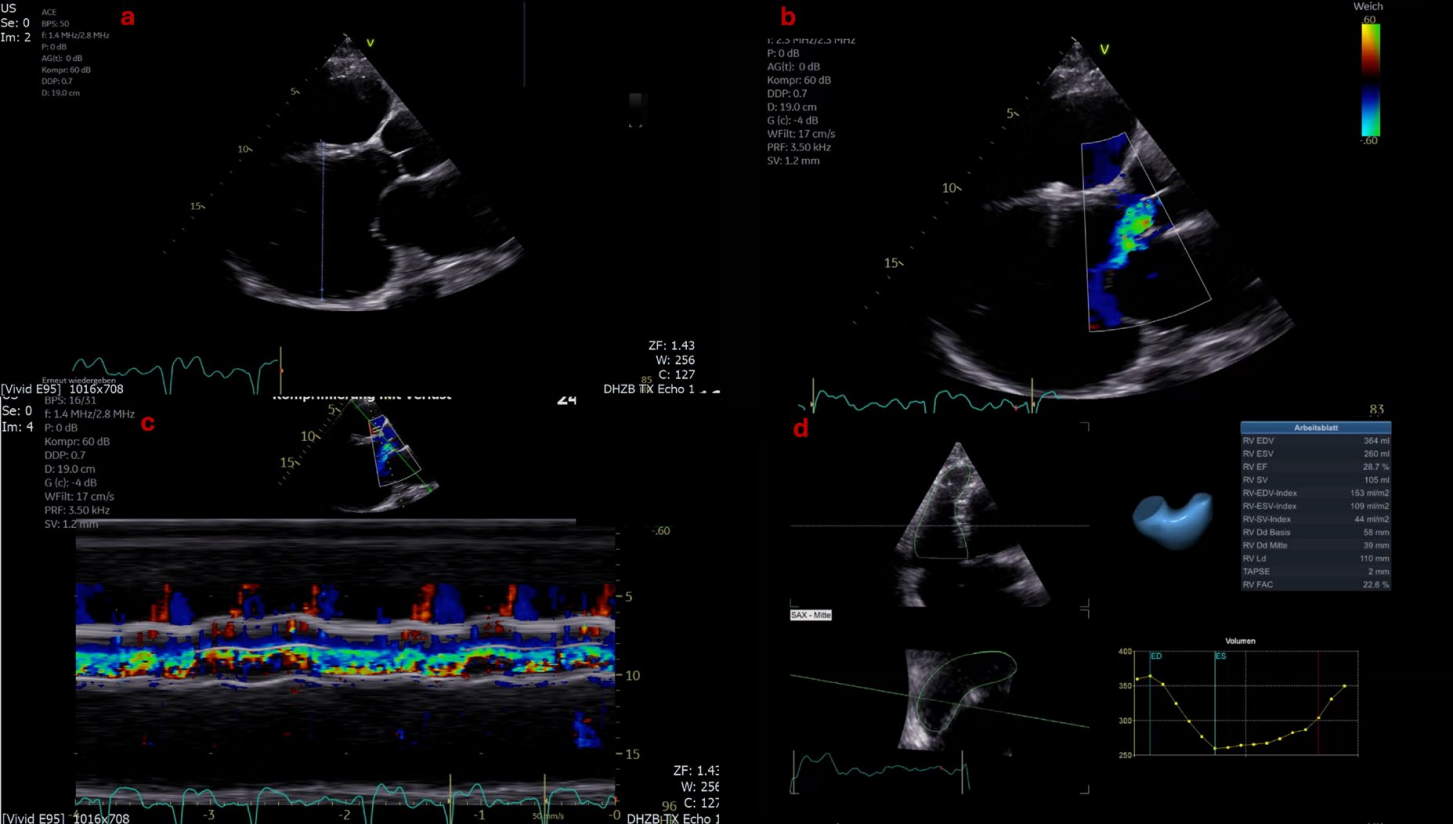
Transthoracic echocardiography: (a) LV dilatation (LVEDD 91 mm) (upper left), (b) significant aortic regurgitation (AR) with eccentric jet toward the mitral valve (upper right), (c) M‐Mode showing diastolic–systolic AR (bottom left), (d) severe RV dysfunction (3D RVEF: 28.7%, FAC: 22.6%) (bottom right). [Color figure can be viewed at wileyonlinelibrary.com]

The current echocardiography (Figure 1, Video S1) revealed LV dilatation, RV dysfunction, and severe AR (diastolic–systolic). A computed tomography (CT) angiography showed a 65% OG stenosis, but the invasive angiography showed no pressure gradient along the OG.

An electrical cardioversion offered no clinical improvement. Large aortic dimensions (annulus: 30.5 mm, Valsalva: 43.9 mm) precluded transcatheter aortic valve implantation (TAVI). Psychosocial factors excluded a heart transplantation.

Despite inotropic and diuretic therapy, low cardiac output syndrome (LCOS) and MOD developed.

**FIGURE 2:**
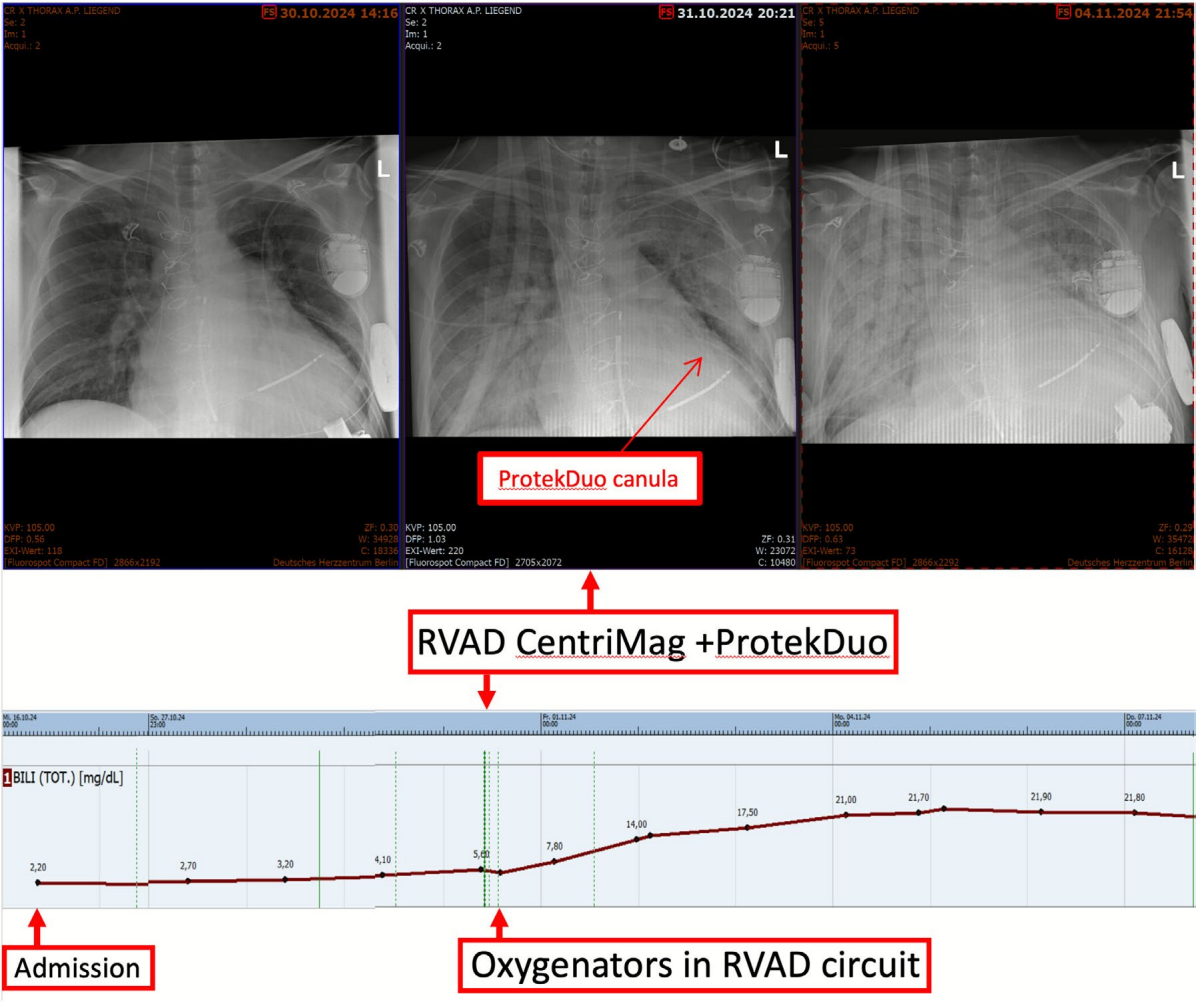
Chest X‐ray (top) and bilirubin course (bottom). After the tRVAD support, the worsening of pulmonary and liver congestion is illustrated. [Color figure can be viewed at wileyonlinelibrary.com]

Increasing the HM3 speed to maximal 9000 rpm displaying a flow of 7.5 L/min did not adequately unload the LV (left ventricular end‐diastolic diameter [LVEDD] from 91 mm to 89 mm) nor improve the hemodynamics. A tRVAD support using Levitronix CentriMag (Levitronix LLC, Waltham, MA, USA) employing a ProtekDuo cannula (LivaNova, London, UK) was used as bridging to durable RVAD and SAVR. Soon the patient developed severe respiratory failure requiring placement of two oxygenators into the RVAD circuit. The chest X‐rays revealed severe pulmonary edema, and lab results showed MOD worsening (creatinine: 2.1 mg/dL, bilirubin 22.9 mg/dL) (Figure 2) and elevated C‐reactive protein (CRP) (280 mg/L). After a suspected infectious acute respiratory distress syndrome (ARDS), a wide‐spectrum antibiotic treatment was initiated. Ten days later, the patient was extubated but pulmonary edema and MOD persisted.

LV overload caused by the tRVAD support combined with AV regurgitant volume and suboptimal forward blood flow was considered a primary cause.

**FIGURE 3:**
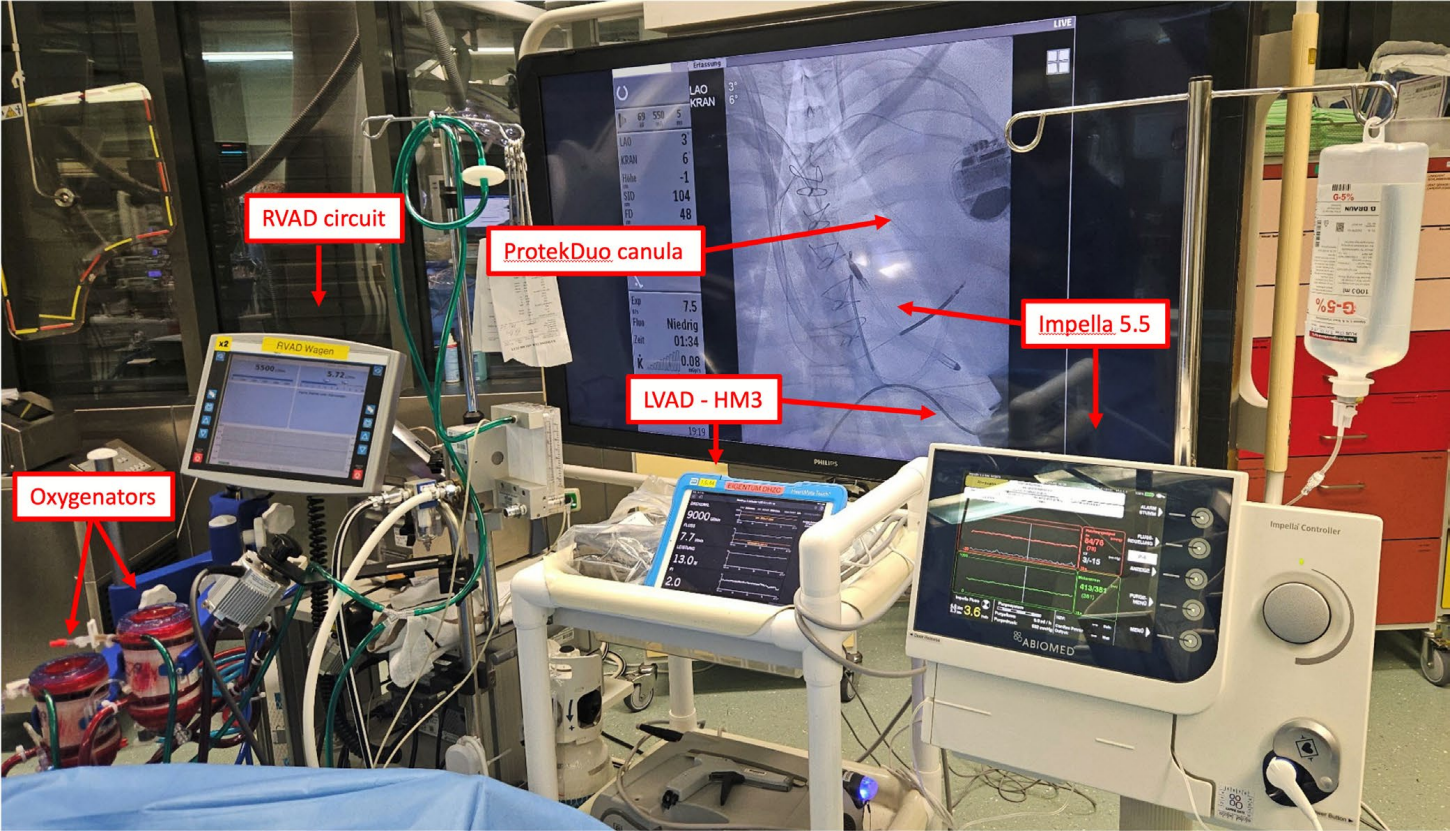
Intraoperative photo of TriVAD (combination of three simultaneously working VAD): 1. temporary right ventricular assist device (RVAD) circuit with Levitronix CentriMag and ProtekDuo cannula combined with two oxygenators (left); 2. left ventricular assist device (LVAD ‐ HeartMate 3) (middle); 3. Impella 5.5 (right). [Color figure can be viewed at wileyonlinelibrary.com]

**FIGURE 4:**
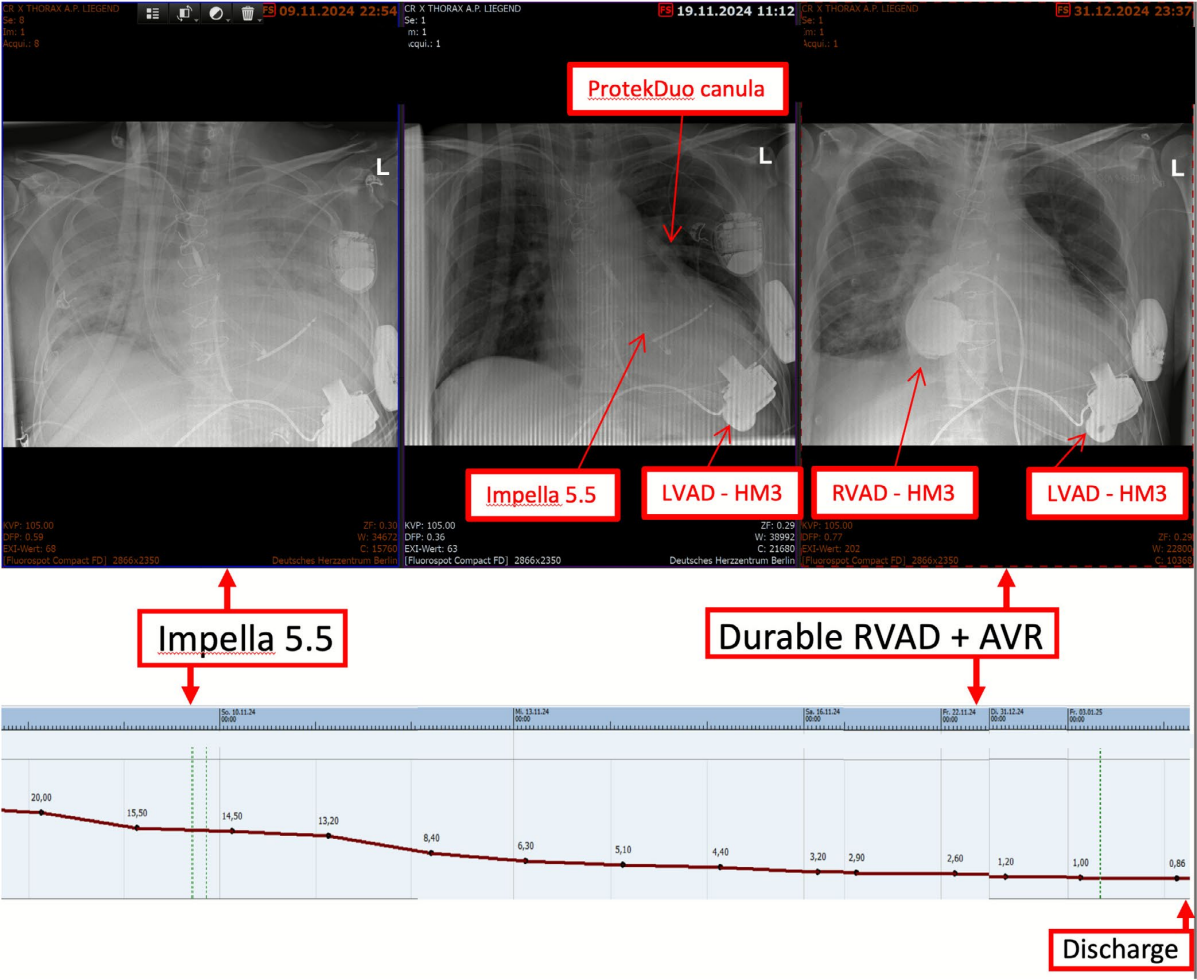
Chest X‐ray (top) and bilirubin course (bottom). After the Impella 5.5 implantation, a noticeable improvement occurred. In the last two chest X‐rays, the TriVAD and the BiVAD combinations (involving HeartMate 3) are indicated. [Color figure can be viewed at wileyonlinelibrary.com]

An Impella 5.5 (Abiomed, Danvers, MA, USA) via the right axillary artery provided additional flow of 5.5 L/min (Figure 3, Video S2). The additional LV unloading normalized the organ function and released the lung edema in the next 3 weeks (Figure 4).

One month later, a durable RVAD with a second HM3 and biological AV (Avalus [29 mm], Medtronic, Minneapolis, USA) was implanted. The patient was discharged home in stable condition 60 days after the final operation.

## 2 Discussion

AR on durable LVAD is a frequent and potentially severe complication [1], potentially requiring surgical or transcatheter replacement [2]. Early AR recognition and treatment is crucial, as unrecognized AR can cause RV failure due to increased RV afterload and effective forward blood flow reduction, both resulting in suboptimal organ perfusion.

AR assessment on LVAD is challenging as conventional parameters (e.g., pHT) have limitations [3]. AR worsening assessment is based on vena contracta, jet width/left ventricle outflow tract (LVOT) ratio, progressive LV dilatation, AR timing in cardiac circle (diastolic and systolic regurgitations indicates at least moderate AR) and RV function evaluation.

In our case, initial AR was at least moderate with preserved RV function. Five months later, AR worsening led to severe RV failure with LCOS and MOD requiring temporary mechanical circulatory support (tMCS) support.

After 10 days of tRVAD support, treatment was suboptimal, as pulmonary edema and MOD persisted. Following misinterpretations delayed the cause recognition:

1. The indicated HM3 flow (7.3 L/min) and measured tRVAD flow (5.3 L/min) led to assumption that the LV was unloaded and the abnormal chest X‐ray was due to ARDS. In reality, the tRVAD caused LV overload and subacute lung edema.
2. The indicated average HM3 flow of 7.5 L/min was enough for organ perfusion. Since the AV regurgitant volume could not be exactly quantified [4], we should assume that the effective forward blood flow (< 4–5 L/min) was inadequate.
3. The venous congestion was due to RV failure which was addressed by tRVAD. In reality, the congestion was caused by the increased LV preload by the tRVAD.

The additional LV unloading by the Impella 5.5 resulted in pulmonary edema and MOD resolution. The durable BiVAD with HM3 and SAVR was performed as final treatment [5].

## 3 Conclusions

The presented clinical case resulted from initial underestimation of the severity of AR and its hemodynamic impact. The unique combination of temporary and durable MCS for the LV (Impella 5.5 and HM3) improved the forward blood flow, eliminating the LV overload, and allowed patient preconditioning till the definitive surgical treatment. To our knowledge, this is the first reported case of Impella support, in a combination of three simultaneously working MCS devices (true TriVAD).

This case also emphasizes the importance of early AR recognition and treatment to prevent RV failure and hemodynamic compromise. In the next case, significant AR should trigger AVR earlier.

## Author Contributions

**Nikolaos Cholevas:** concept/design, data analysis/interpretation, drafting article, critical revision of article, approval of article, statistics, data collection. **Felix Schoenrath:** critical revision of article, approval of article. **Nicolas Merke:** data analysis/interpretation, drafting article, critical revision of article, approval of article, data collection. **Denis Fries:** critical revision of article, approval of article. **Marcus Mueller:** critical revision of article, approval of article. **Johanna Mulzer:** critical revision of article, approval of article. **Evgenij Potapov:** concept/design, data analysis/interpretation, drafting article, critical revision of article, approval of article, statistics, data collection.

## Funding

The authors have nothing to report.

## Disclosure

The authors have nothing to report.

## Conflicts of Interest

The authors declare no conflicts of interest.

## Supporting information


**Video S1:** Echo loops indicating: (a) significant AR with eccentric jet towards the mitral valve, (b) significant AR in transesophageal echo, (c) severe tricuspid regurgitation (TR), (d) severe RV dysfunction with 3D evaluation.

**Video S2:** The TriVAD combination: Impella 5.5 implantation on the top of LVAD and tRVAD (ProtekDuo canula visualized).

## Data Availability

The data that support the findings of this study are available on request from the corresponding author. The data are not publicly available due to privacy or ethical restrictions.
